# Genome-scale identification of *Caenorhabditis elegans *regulatory elements by tiling-array mapping of DNase I hypersensitive sites

**DOI:** 10.1186/1471-2164-10-92

**Published:** 2009-02-25

**Authors:** Baochen Shi, Xiangqian Guo, Tao Wu, Sitong Sheng, Jie Wang, Geir Skogerbø, Xiaopeng Zhu, Runsheng Chen

**Affiliations:** 1 Bioinformatics Laboratory and National Laboratory of Biomacromolecules, Institute of Biophysics, Chinese Academy of Sciences, Beijing 100101, PR China; 2 Department of Molecular Physiology & Biological Physics, School of Medicine, University of Virginia, 1300 Jefferson Park Ave, Charlottesville, Virginia 22908, USA; 3 Graduate School of the Chinese Academy of Science, Beijing 100080, PR China

## Abstract

**Background:**

A major goal of post-genomics research is the integrated analysis of genes, regulatory elements and the chromatin architecture on a genome-wide scale. Mapping DNase I hypersensitive sites within the nuclear chromatin is a powerful and well-established method of identifying regulatory element candidates.

**Results:**

Here, we report the first genome-wide analysis of DNase I hypersensitive sites (DHSs) in *Caenorhabditis elegans*. The data was obtained by hybridizing DNase I-treated and end-captured material from young adult worms to a high-resolution tiling microarray. The data show that *C. elegans *DHSs were significantly enriched within intergenic regions located 2 kb upstream and downstream of coding genes, and also that a considerable fraction of all DHSs mapped to intergenic positions distant to annotated coding genes. Annotated transcribed loci were generally depleted in DHSs relative to intergenic regions, but DHSs were nonetheless enriched in coding exons and UTRs, whereas introns were significantly depleted in DHSs. Many DHSs appeared to be associated with annotated non-coding RNAs and recently detected transcripts of unknown function. It has been reported that nematode highly conserved non-coding elements were associated with cis-regulatory elements, and we also found that DHSs, particularly distal intergenic DHSs, were significantly enriched in regions that were conserved between the *C. elegans *and *C. briggsae *genomes.

**Conclusion:**

We describe the first genome-wide analysis of *C. elegans *DHSs, and show that the distribution of DHSs is strongly associated with functional elements in the genome.

## Background

With full genome sequences available for a number of species, it is now possible to extract further information on how the genome is functionally organized. Identification of regulatory elements of both coding and non-coding genes is therefore a major challenge of the post-genomics era. *Caenorhabditis elegans *is an important multicellular model organism for research on functional genomics and developmental biology, and has delivered a wealth of information with relevance also to research on human diseases and aging. *C. elegans *was the first to metazoan to have its genome sequenced [1], however, *C. elegans *genome annotation and molecular functional research have thus far mainly focused on the transcribed part of the genome. Today a central challenge is to obtain a complete and accurate identification of the gene regulatory elements in the *C. elegans *genome. However, so far no genome-wide analysis of the *C. elegans *regulatory elements has been reported.

At the large-scale chromatin level, nuclease hypersensitive sites are open windows that allow enhanced access for trans-acting factors to cis-regulatory DNA elements. DNase I is an enzyme that preferentially digests nucleosome-depleted DNA, whereas tightly packaged chromatin is more resistant to cleavage. Historically, mapping DNase I hypersensitive sites (DHSs) by Southern blotting has been the standard method for identifying the location of functional regulatory elements such as promoters, enhancers, silencers, insulators and locus control regions [2]. Unfortunately, this method is time-consuming and cannot readily be applied simultaneously on a full genome-scale. Collins and coworkers reported the first genome-wide library of human DHSs using the massively parallel signature sequencing (MPSS) [3], showing that approximately 80% of DHSs uniquely map within annotated regions of the genome believed to contain regulatory elements. They also found that most DHSs identified in CD4^+ ^T cells were also DNase I hypersensitive in five other cell lines. Recently, two groups have also reported high-throughput analyses of DHSs in 1% of the human genome using tiling arrays (the ENCODE project; [4,5]). Collins *et al *[3] further found that there was an enrichment of DHSs detected within the 2 kb upstream and downstream of genes, and in first exons, first introns, CpG islands and highly conserved regions. In contrast, DHSs were significantly depleted in non-first exons and introns, and in distal intergenic regions [4]. Sabo *et al *found that DHSs were enriched in introns and in regions proximal to transcription start sites (TSSs) and transcription termination sites (TTSs), and were depleted in distal intergenic regions [5].

Here, we describe the first genome-wide analysis of *C. elegans *DHSs, in which DNase I-treated and end-labeled genomic DNA was hybridized to a tiled microarray covering the entire genome. The identified DHSs constitute regulatory elements candidates for coding and non-coding genes, and improves our understanding of the regulation of gene expression at the chromatin structure level.

## Methods

### Preparation of DNase I-treated DNA

The development stages of *Caenorhabditis elegans *(strain N2) were observed by periodically scoring sizes of worms cultivated at 20°C under a microscope. To obtain synchronized young adult worms, gravid worms were treated with lysis solution (NaClO 10 mL, NaOH 2 mL, H_2_O 8 mL), the collected embryos incubated in M9 buffer for more than 7 hrs at 20°C with shaking, and then fed OP50 bacteria at 20°C for about 54 hrs. Subsequently, synchronized worms were treated by shaking in S buffer with 25 uM floxuridine (FUdR, Sigma) for 8–9 hrs at 20°C. Floxuridine is a competitive inhibitor of thymidilate synthetase and blocks DNA replication without any apparent effect on the vitality and longevity of the worms [6,7].

Worm nuclei were isolated with the Nuclei Isolation Kit (Sigma) according to the instructions of the manufacturer. In the nucleus, most genomic DNA is wrapped around and protected by protein complexes, leading to the formation of regularly spaced nucleosomes. Regions of intact nuclei genomic DNA without nucleosome formation (i.e., nucleosome-free regions) can be digested with high concentrations of DNase I. In this study, we treated intact nuclei with four concentrations (0, 240, 480 or 800 U/ml) of DNase I (Fermentas, 1 U/μl) for 5 min at 37°C. One control sample was incubated on ice without DNase I for the same period of time. The DNase I digestions were terminated by adding an equal volume SDS buffer (4 ml SDS buffer + 8 ul 10 mg/ml RNase A), and incubated at 55°C for more than 8 hrs followed by addition of Proteinase K to a final concentration 25 mg/ml. The samples were then extracted with phenol-chloroform, precipitated with ethanol, and digested with RNase A/T1 mix (Fermentas, 2 mg/ml of RNase A and 5000 u/ml of RNase T1) at 37°C for 30 minutes. DNA from the RNase-treated samples was extracted with phenol-chloroform, ethanol precipitated, washed with 70% ethanol, and dissolved in ddH_2_O. Finally, we selected samples treated with two concentrations (240 U/ml and 480 U/ml DNase I) to be prepared for tiling array assays along with the control sample.

The naked DNA control sample was obtained by directly digesting extracted intact nuclei with Proteinase K, followed by treatment with DNase I. Without the protection of protein complexes, naked genomic DNA is far more susceptible to DNase I digestion, this sample was treated with much lower concentrations of DNase I (0.05 U/ml or 0.1 U/ml) at 37°C for 5 min, and aliquots from the two DNase I-treatments were mixed to generate pools of random control fragments. Naked DNA was digested with multiple concentrations of DNase I to rule out sequence-based bias of DNase I digestion [4]. The fragment length distribution of the DNase I-treated naked DNA sample was similar to that of the DNase I-treated chromatin-specific DNA.

### Tiling microarray assay

After treatment with T4 Polynucleotide Kinase (NEB) the DNase I-treated fragments were blunt-ended with Klenow DNA Polymerase (NEB), purified with the QIAquick Nucleotide Removal Kit (Qiagen), and ligated to the biotinylated Adaptor-I (sequence available in Additional file 1) with T4 DNA ligase (NEB). The ligated products were purified on a MicroSpin S-400 spin column (GE Healthcare), sonicated to obtain fragments with a median length of 500 bp, purified with biotin-streptavidin interaction magnetic beads (Dynal), phosphorylated and blunted-end as above, and ligated to Adaptor-II (sequence available in Additional file 1). Adapter-ligated DNase I-treated fragments attached to Dynal beads were amplified by PCR with Platinum Taq DNA Polymerase (Invitrogen). The PCR products were purified with the Gel and PCR Clean-up System (Promega), end-labeled using the GeneChip Whole Transcript Double-Stranded DNA Terminal Labeling Kit (Affymetrix), and the efficiency of the labeling procedure was assessed with a gel-shift assay.

DNase I-treated and control samples were hybridized to the Affymetrix GeneChip^® ^*C. elegans *Tiling 1.0R Array, which contains ~3.2 million perfect match/mismatch probe pairs tiled through the Watson strand of the entire non-repetitive *C. elegans *genome. The probes are tiled at an average distance of 25 bp, as measured from the central position of adjacent 25-mer oligonucleotide probes. Sequences used in the design of this array were based on WormBase release WS140 assembly (26 Mar, 2005) [8]. The raw array data (CEL files) are available on request, and the signal intensity distribution for each array is shown in Additional file 1.

### Validation of DHSs by real-time PCR

In several previous studies, real-time PCR has been used in the validation of DHSs in the human genome and in the quantitative analysis of DNase I-hypersensitivity of the mouse beta-globin LCR [3,4,9]. Briefly, primer sets designed to produce fragments covering DHSs from all three mutually exclusive categories were used to amplify genomic DNA from samples that were either undigested or treated with 240 U/ml and 480 U/ml DNase I (primer sequences are available on request). In the DNase-treated samples, a valid DHS is expected to require an increased number of cycles (ΔCp) to generate the same amount of PCR product as in the undigested sample. Several previous studies from both microarray and high-throughput sequencing data have shown that 95% of the primer sets surrounding random selected regions of the genome displayed ΔCp values of less than two, and a threshold of ΔCp > 2 has been generally accepted for validation of DHSs, as ΔCp > 2 in principle reflect a four-fold reduction in DNA concentration [3,4]. In this study, we followed this definition and any primer set that generated a real-time PCR ΔCp value above 2 was considered as a true positive. All PCR reactions were performed on a LightCycler 2.0 instrument (Roche).

### Computational analyses

Raw tiling microarray data analysis and DHSs identification were performed by implementing the Affymetrix Tiling Analysis Software (TAS, version 1.1.02). Briefly, quantile-normalization were performed on the biological replicates within the treatment and control groups respectively [10], and the normalized intensities were then scaled to set the median intensity of 128 for each array. As for each perfect matched (PM) probe in the tiling array there also exist a mismatched (MM) probe, the signal intensities for each probe on both the control and treatment tiling arrays were transformed into a value S = log2 (max (PM-MM, 1). A non-parametric Wilcoxon signed-rank test was applied to the S-values from the treatment and control arrays in a sliding window across the genome, testing whether the distribution of the S-values for the treated samples is shifted up relative to that of the control data [11]. The size of the sliding window was set to 500 bp, which corresponded to the median fragment length of the DNase I-treated sample before PCR enrichment. The window was centered at the genomic coordinate of each oligonucleotide probe, and a p-value measuring the likelihood that the region is a DHS was assigned to the probe. The p-value was computed using a Wilcoxon paired signed rank test comparing test signal against a reference signal for all oligos in the window, and a p-value < 0.01 designated a positive probe. A DHS was subsequently defined as two or more consecutive positive probes whose central positions were separated by less than 50 bp.

The *C. elegans *genome annotation and sequence data were downloaded from WormBase (release WS140) [8]. A Monte Carlo simulation was performed to determine the distribution bias of DHSs relative to annotated genomic elements by testing the null hypothesis of no difference between the distribution of DHSs and random selected regions relative to annotated genomic elements [4]. In the simulation, which was repeated 1000 times, genomic regions corresponding in length, number and chromosomal distribution to the DHSs were randomly selected from the WormBase WS140 release of the genome. The mixed-staged *C. elegans *nucleosome core position data were obtained from Johnson *et al *[12], and the conservation analysis between *C. elegans *and *C. briggsae *was obtained from Kent and Zahler [13]. The *C. elegans *non-coding RNA data was obtained from WormBase annotations [8] and our own studies [14,15]. The *C. elegans *gene expression datasets were obtained from the Genome B.C. *C. elegans *Gene Expression Consortium . We used the Pearson correlation coefficient to describe the relationship between the distributions of DHSs and coding genes along each chromosome. The Pearson correlation coefficient takes the form:

$$\gamma_{xy}=\frac{\sum X_{i}Y_{i}-\left(\sum X_{i}\sum Y_{i}\right)/n}{\sqrt{\sum X_{i}^{2}-{\left(\sum X_{i}\right)}^{2}/n}\sqrt{\sum Y_{i}^{2}-{\left(\sum Y_{i}\right)}^{2}/n}}$$

where Xi and Yi are the number of DHSs and genes, respectively, in one Mb non-overlapping windows along each chromosome; a γ_*xy *_value close to 1 meaning that DHSs and genes have a consistent distribution along the chromosome.

## Results

### Tiling array assays and validation

**Figure 1:**
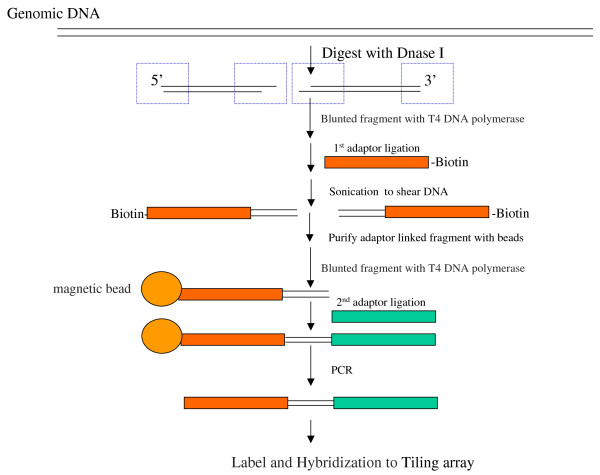
**Protocol outline for the genome-scale mapping of *C. elegans *DHSs by tiling microarray analysis**.

**Figure 2:**
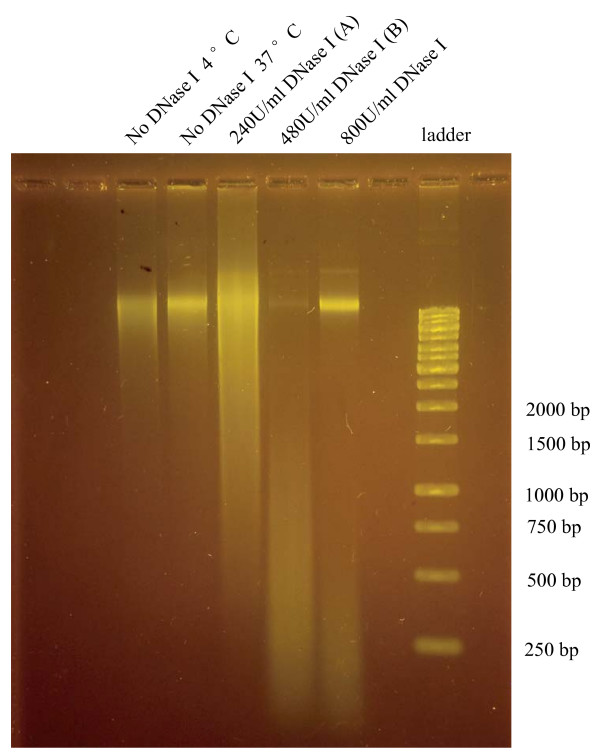
**Gel electrophoresis of DNase I – digested nuclear DNA**.

The protocol for the genome-scale mapping of *C. elegans *DNase I hypersensitive sites (DHSs) by the tiling microarray is summarized in Figure 1. The Affymetrix *C. elegans *Tiling 1.0R array contains ~3.2 million 25-mer oligonucleotide probe pairs covering the Watson strand of the entire non-repetitive genome at an average resolution of 25 bp. Synchronized worms in the young adult (YA) stage were treated with floxuridine (FUdR) for more than 8 hours to reduce the background signal from reproduction, without any apparent effect on vitality and longevity [6,7]. Extracted nuclei were digested with different concentrations of DNase I (Figure 2), and samples treated with 240 and 480 U/ml (along with DNA from untreated nuclei) were applied to the tiling assays. The entire procedure was replicated after an interval of about one month, and quantile-normalization was performed on the biological replicates within treatment and control groups [10]. To identify probes that are significantly (p < 0.01) shifted up relative to the control data, a non-parametric Wilcoxon signed-rank test was applied to the data from the treatment and control arrays in a sliding 500-bp window across the genome. A DHS was defined as two or more consecutive positive probes whose central positions are separated by less than 50 bp. Estimated from the negative control probes designed within the Affymetrix microarray, this approach resulted in false positive rates of 0.3% and 0.14% for the 240 U/ml and 480 U/ml DNase I-treated samples from the array readout, respectively. We defined three mutually exclusive DHSs categories; DHSs identified in both samples (875 DHSs), and DHSs only present in one of the two samples treated with either 240 U/ml DNase I (3953 DHSs) or 480 U/ml DNase I (2267 DHSs). The coordinates for all DHSs detected by tiling arrays can be downloaded at .

Real-time PCR was used to validate the microarray data, and DHSs were considered as true positives when the number of additional cycles required to achieve threshold amplification from DNase I-treated nuclear DNA (compared with non-digested control genomic DNA; ΔCp) was higher than two [3,4]. The fractions of validated samples were ~86%, 77% and 91% for the three DHSs categories, respectively (Table 1).

**Table 1:** Validation of DHSs by real-time PCR

DHS category	DHSs tested	ΔCp > 2.0	Fraction
A	13	10	76.9%
B	11	10	90.9%
AB	14	12	85.7%

DHSs were regarded as true positives when the corresponding real-time PCR assay ΔCp was larger than 2 [3,4]. DHS category denotes DHSs that were present only in samples digested with (A) 240 U/ml or (B) 480 U/ml DNase I, or present in both samples (AB).

### Genomic distribution of DHSs within the annotated genome

**Figure 3:**
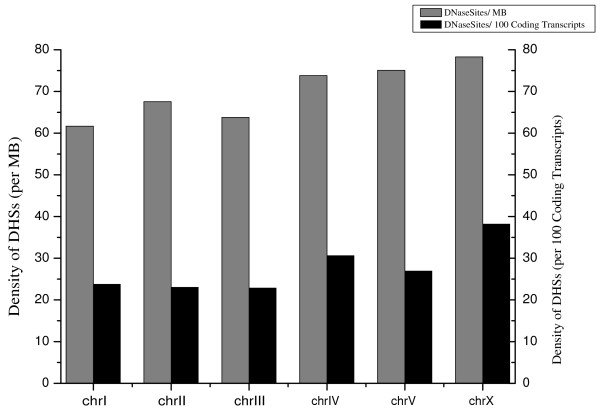
**Chromosomal DHS densities**.

The average DHSs length was 121 bp, with maximum and minimum lengths ranging from 46 bp to 754 bp (see Supplementary Figure S2 in Additional file 1 for the DHS length distributions). The locations of all 7095 DHSs were mapped to the *C. elegans *genome (WormBase WS140) [8]. The density of DHSs was slightly larger on the chromosome X than on the other chromosomes. This difference was similar to the distribution of highly conserved non-coding elements (CNEs) in the *C. elegans *genome [16], and could not be entirely explained by the density of annotated coding genes on chromosomes X, as the number of DHSs per 100 annotated coding genes were also higher for chromosomes X than for autosomal chromosomes (Figure 3).

**Figure 4:**
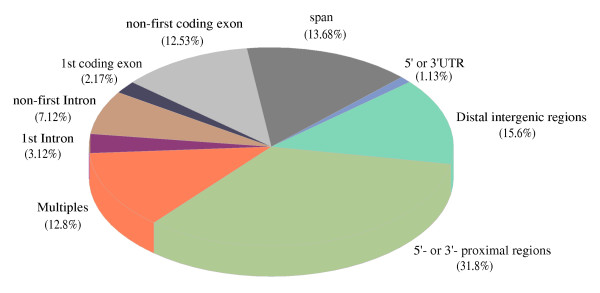
**DHS genomic locations**. "Proximal" and "nearby" have the same meaning, and refer to locations within 2 kb from the transcriptional start sites (TSSs) or transcription termination site (TTSs) of the nearest coding genes. "Distal" intergenic locations correspondingly refer to locations more than 2 kb from a TSS or TTS. "Multiples" refers to DHSs located within loci annotated with more than one coding transcript, and "span" means DHSs spanning junctions between exons and introns.

A statistical simulation (Monte Carlo simulation) was performed to determine the distribution bias of DHSs relative to annotated genomic elements. It has been estimated that approximately 60% of the total *C. elegans *genome is transcribed as protein-coding genes based on the annotation of WormBase WS140 [8]. In this study, we found that *C. elegans *DHSs were significantly depleted in intragenic regions (p-value < 0.001, see Supplemental Table S1 in Additional file 1). A supplemental table listing the confirmed coding genes with nearby DHSs was provided in Additional file 2. Approximately 40% of the *C. elegans *DHSs map unequivocally within the bounds of protein coding loci (Figure 4). Around 2.17% of all DHSs were located to the first coding exons, which represent an enrichment compared to the random set (p-value < = 0.053). In contrast to human DHSs, which are significantly depleted in internal (i.e. non-first) exons [4], there appear to be no statistical differences in DHS locations with respect to exon positions in the nematode. The 10.2% DHSs found in intronic locations represent, on the other hand, a significant depletion compared to the random set (p-value < 0.001), suggesting that intragenic regulatory elements in *C. elegans *are predominantly located in coding sequence. The few percent of the DHSs residing within 5' and 3' UTRs represented a slight enrichment over a random distribution (p-value < = 0.057). In addition to the 40% of the DHSs with a certain genic location, 12.8% of DHSs mapped to loci annotated with several different and/or overlapping transcripts, and the precise genomic status of these DHSs could not be determined.

**Figure 5:**
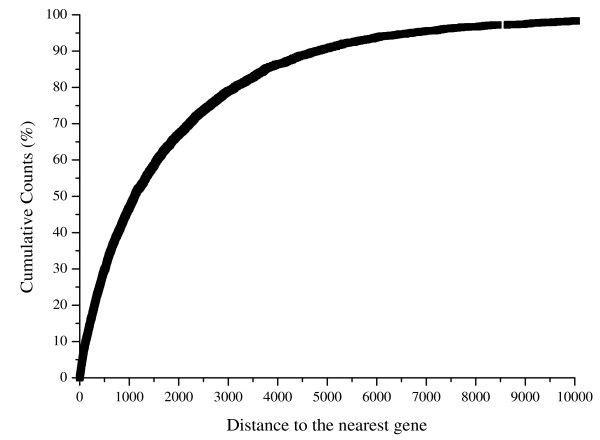
**Distribution of intergenic DHSs relative to transcriptional start sites (TSSs) or transcription termination sites (TTSs) of the nearest coding genes**.

In accordance with previous studies [5], about one half of the DHSs map to intergenic regions. A large fraction (67.1%) of the intergenic DHSs located within 5'- or 3'-proximal regions (i.e. 2 kb upstream or downstream) of coding genes (Figure 5), which represent a significant enrichment (p-value < 0.005) over the random set. This is also consistent with the previous observation that DHSs tend to be enriched at regions expected to harbor active regulatory elements [17]. On the other hand, one third of intergenic DHSs mapping more than 2 kb away from any known coding genes also represent a slightly higher fraction than would be expected from the random distribution (p-value < 0.067). This suggests that some transcriptional regulatory information is located far from currently annotated genes, however, the targets of such regulatory elements are difficult to determine.

The DHSs commonly occur in DNA sequences that were conserved between *C. elegans *and *C. briggsae*. Approximately 48% of DHSs were located within evolutionarily conserved regions across the whole genome including the coding regions, non-coding regions and intergenic regions [13], a percentage significantly higher than for the randomly selected regions (p-value < = 0.001, see Supplemental Table S2 in Additional file 1). In particular, distal intergenic DHSs show a statistically significant (p-value < 0.038) tendency to fall in regions that are conserved between the two nematode genomes. We also found that a high fraction of the DHSs (68.8%) were located within nucleosome-free regions of the mixed-staged *C. elegans *nucleosome core positioning landscape [12], suggesting that nucleosome-free regions are generally more DNase I sensitive.

### The relationship between DHSs and gene categories

**Figure 6:**
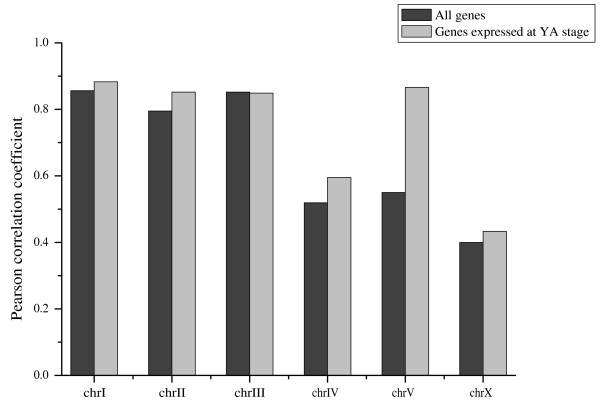
**DHS distribution relative to gene expressional characteristics**. Relationship between the distributions of DHSs and genes expressed at young adult stage. The Pearson correlation coefficients were calculated between the frequency of DHSs and YA genes in 1 Mb non-overlapping windows along each chromosome.

To analyze whether the DHSs were associated with nearby gene expression, we used data from the Genome B.C. *C. elegans *Gene Expression Consortium to identify genes expressed at the young adult stage (henceforth called YA genes; see Additional file 1 for details). We used these data to calculate Pearson correlation coefficients (PCCs) between the frequency of DHSs and annotated genes within one Mb non-overlapping windows along each chromosome. For most chromosomes, particularly in chromosome V, the distribution of DHSs correlated more strongly with the distribution of YA genes than with that of all annotated coding genes (Figure 6). It has been reported that genes in the vicinity of DHSs show increased levels of gene expression [3,4]. To further explore the relationship between DHSs and nearby gene activity, we calculated correlations between the distance of a DHS center to its nearest gene and the expression level of that gene (Supplementary Figure. S10 in Additional file 1). As expected, YA genes with nearby DHSs (2 kb upstream or downstream) were likely to have higher expression levels than genes located more than 2 kb from a detected DHS. However, consistent with previous studies [4], the presence of a DHS does not necessarily imply an elevated expression level of the nearest gene.

We also examined the distribution of DHSs relative to known non-coding RNAs (ncRNAs), based on annotations in WormBase (WS140) [8] and data from our lab [14]. We found DHSs located within or proximal (500-bp upstream or downstream) to 66 known ncRNAs including tRNAs, snoRNAs, microRNAs, snRNAs and snlRNAs, suggesting that a number of DHSs may possibly represent elements involved in transcriptional regulation of non-coding RNA genes. Nonetheless, the frequency of DHSs nearby known ncRNAs was slightly less (p-value < 0.06) than the random set (Supplementary Figure. S5 and Figure. S8 in Additional file 1). We also asked whether the occurrence of DHSs was correlated with small transcripts of unknown function (TUFs) identified by the whole-genome tiling microarray [15]. We found about one third of the intronic DHSs surround TUFs representing a significant depletion (p-value < 0.001); and only a minor fraction (~6%) of the intergenic DHSs were situated nearby TUFs, which also represented a depletion compared to the random set (p-value < 0.005). These observations indicated that DHSs may possibly be less important as regulatory elements for non-coding RNA genes than for coding genes. In addition, some DHSs were located within or close to 196 pseudogenes (Additional file 3). The *Caenorhabditis elegans *genomic organization is particular in that a high fraction (15%) of genes are found in operons from which a polycistronic primary transcript is processed to monocistronic mRNAs [18]. We found that 16.3% of intragenic DHSs map within the bounds of 380 operons. Although this does not represent any significant difference in DHS frequency between operonic genes and non-operonic genes (Supplementary Figure S7 in Additional file 1), the fact that operons have internal DHSs may indicate the existence of particular internal regulatory elements involved in operon expression [19].

## Discussion

Compared to the amount of information that has been accumulated on gene expression, our understanding of gene regulation in metazoans is still limited. In this study, we report the first genome-wide mapping of DNase I hypersensitive sites in the multicellular model organism *Caenorhabditis elegans *by a high-resolution tiling microarray. Similar to the DNase-chip method developed by Crawford *et al*. [4], DNA fragments flanking DNase I-cleavage sites were captured by ligation to biotinylated adapters and amplification by PCR. Since replicating DNA forks are susceptible to DNase I digestion, Crawford *et al. *[4] used the non-replicating CD4^+ ^T cells to reduce background. Here, we treated synchronized young adult hermaphrodite worms with floxuridine (FUdR) to block cell division, thereby further reducing the levels of DNA replication background [6,7]. In the study, we actually only identify DHSs that are common in the mixture of all cell types at the young adult stage. However, different cell types within worms could have drastically different gene expression and chromatin profiles. Subsequent studies of DHSs profiles from primary tissues and at various development stages should therefore further increase our understanding of the dynamic expressional regulation at the chromatin structure level in the nematode.

**Figure 7:**
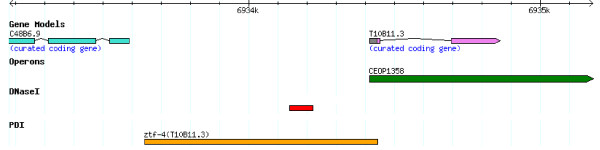
**An example of a DHS located within the known promoter region of a coding gene expressed at the adult stage**.

Consistent with previous regulatory element studies in human genome [20,21], DHSs were found throughout the *C. elegans *genome. We found that about one half of the DHSs map to intergenic regions, and that two thirds of the intergenic DHSs were located within upstream or downstream proximal regions of coding genes. In a recent study on human transcriptional promoters and enhancers, approximately 70% of putative distant regulatory elements detected by ChIP-on-chip assays in HeLa cells overlapped with DHSs [22]. We found DHSs located within eight of *C. elegans *known coding gene promoter regions identified by high-throughput yeast one-hybrid (Y1H) assays [23]. For example, Figure 7 showed a DHS located within the promoter region of a gene expressed at the adult stage (T10B11.3), which is a member of the Zinc finger Transcription Factor family [8]. We also found that one-third of the intergenic DHSs map to regions more than 2 kb away from coding genes, suggesting that these may represent long-distance regulatory elements candidates; however, as a considerable fraction of the intergenic DHSs are located nearby putative ncRNA loci, there is also the possibility that these DHSs may be regulatory elements targeting not yet identified non-coding genes.

We found that the frequency of intronic DHSs is significantly less than would be expected based on the amount of genomic sequence occupied by introns, but about one-fourth of all genic DHSs are nonetheless located in introns. A reasonable expectation would be that these elements contain regulatory activity targeting the host gene [24,25]. On the other hand, it has also been demonstrated that long-range regulatory element may be located in introns of very distant genes; for example, the enhancer of the SHH gene was found within an intron of a gene located one Mb away in the human genome [26]. In addition, regulatory elements of non-coding RNAs have been reported in introns [14], and analysis of the genomic distribution of DHSs with respect to non-coding RNA loci showed that one third of the intronic DHSs surround known or putative small ncRNA loci [14,15].

**Figure 8:**
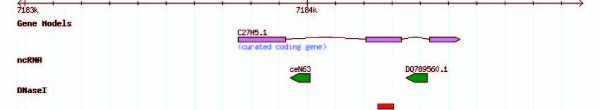
**An example of a DHS located in an exon between two intronic snoRNAs**.

Consistent with previous studies reported in the human genome, DHSs in the *C. elegans *genome were enriched in the first exons that were considered as parts of the core promoters [4,17]. In contrast, a considerable and significantly enriched proportion of the DHSs is also found in internal exons in the *C. elegans *genome. Such DHSs have been suggested to play a role in alternative splicing of the host gene [25], but could also be transcription factor binding sites that regulate the host gene [24,27-29]. Compared to intergenic and intronic DHSs, only a small fraction (10%) of the exonic DHSs is located nearby non-coding RNAs, including 27 internal exonic DHSs nearby known or putative small ncRNA loci. For example, a DHS located in the second exon of a gene (C27H5.1) resides less than 50 bp downstream of the snoRNA (DQ789560.1) locus and less than 240 bp upstream of another snoRNA (CeN63) locus [14,30] (Figure 8).

DHSs were also located within or close to pseudogenes. These DHSs could be regulatory elements of nearby coding genes, but do also raise the possibility that some assumed pseudogenes are active as non-coding genes. Nucleosomes have been observed to be depleted on active regulatory elements throughout the yeast genome [31,32]. In *C. elegans *genome, we also found that approximately 70% of DHSs were found in nucleosome-free regions of mixed-stage worms. It has been reported that nematode highly conserved non-coding elements (CNEs) were associated with cis-regulatory elements [16], and DHSs, particularly distal intergenic DHSs, were also observed to significantly tend to fall in regions that are conserved between the two nematode genomes. Future studies aimed at conserved DHSs will help to determine what type of functional elements these regions may represent.

When exploring the relationship between DHSs and the expression of nearby coding transcripts we found that the chromosomal distributions of DHSs were more strongly correlated to the distribution of genes expressed at the young adult stage than to the general distribution of annotated coding genes (Figure 6). This was most pronounced for chromosome V, despite that the ratio of genes expressed at the YA stage is lower on chromosome V than on other chromosomes (Supplemental Table S3 in Additional file 1). Genes nearby DHSs were more likely to have elevated gene expression; nonetheless, some highly expressed genes did not have any nearby DHSs. This could owe to a variety of reason, one of which might be that DHSs are associated not only with various functional regulatory elements, but could also be linked to other epigenetic signals and non-regulatory structural elements that contribute to chromatin organization [2]. This implies that the relationship between DHSs appearance and the expression of their neighboring coding genes may be not straightforward. We also found that not all DHSs detected after treatment within the lower concentrations of DNase I were observed after treatment with higher concentrations of DNase I, and vice versa. The reasons for this are not clear, whereas the most likely reason for this is stochastic variation in the material or the amplification process, we cannot exclude the possibility that sites may differ in their sensitivity to different DNase I concentrations. There is also the possibility that variation in the completeness of digestion caused by variation in DNase I concentration could lead to sequence-based bias of DNase I digestion [4] and sequence-based differences in amplification or hybridization to the tiling microarray. The latter might be particular true with respect to DHSs located within or adjacent genomic repeat regions, as such sequences are generally excluded from the tiling microarray design. Thus, high-throughput sequencing methods would be a valuable complementary strategy for further identification of DHSs in the *C. elegans *genome.

## Conclusion

In conclusion, we report the first genome-wide mapping of DNase I hypersensitive sites in the multicellular model organism *Caenorhabditis elegans *by a high-resolution tiling array. Combined with the corresponding progresses in the modENCODE project , further studies of DHSs profiles at various development stages and from primary tissues will undoubtedly throw more light on the function of the metazoan genome.

## Authors' contributions

BS, TW and XG conceived and designed the experiments, analyzed data and prepared the manuscript. SS contributed to the experimental design; JW and XZ assisted in the bioninformatic analysis; GS contributed to the writing and discussion of the paper. RC guided and supervised the whole project. All authors have read and approved of the final manuscript.

## Supplementary Material

Additional file 1 **Supplemental Data.** The data provided the supplemental statistical analysis about genomic distribution of DHSs within the annotated genome and the relationship between DHSs and gene categories. Click here for file

Additional file 2 **Additional_table-S1.** This table includes confirmed coding transcripts with nearby DHSs (from 2 kb upstream to 2 kb downstream). Click here for file

Additional file 3 **Additional_table-S2.** This table includes Pseudogenes with nearby DHSs (from 2 kb upstream to 2 kb downstream). Click here for file
