# Supplementary material for: Genome-scale identification of Caenorhabditis elegans regulatory elements by tiling-array mapping of DNase I hypersensitive sites
Source: BMC Genomics. 2009 Feb 25;10:92. doi: 10.1186/1471-2164-10-92 (PMC2651899; doi:10.1186/1471-2164-10-92)
Supplement: Additional file 1 — Supplemental Data. The data provided the supplemental statistical analysis about genomic distribution of DHSs within the annotated genome and the relationship between DHSs and gene categories. [file 1471-2164-10-92-S1.doc]

Supplemental Data

Genome-scale identification of *Caenorhabditis elegans* regulatory elements by tiling-array mapping of DNase I hypersensitive sites

Additional files and supplemental Data can also be found online at http://bioinfo.ibp.ac.cn/dnase/.

Supplemental Document 1:


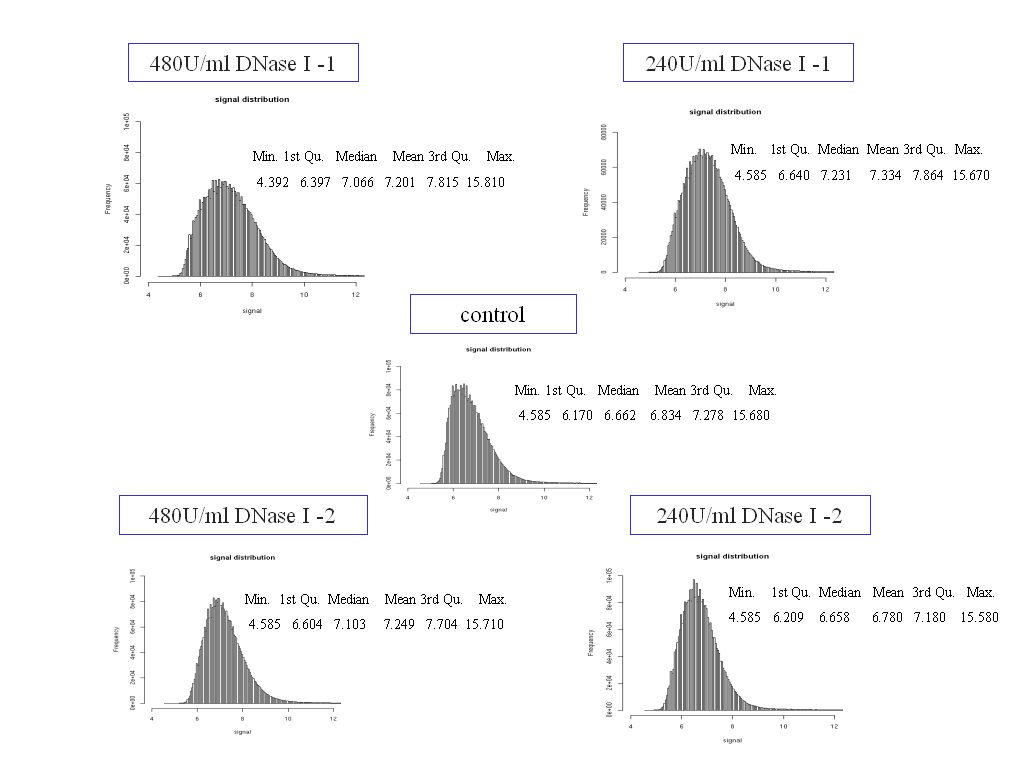


Supplementary Figure.S1 Signal intensity distribution for each array.


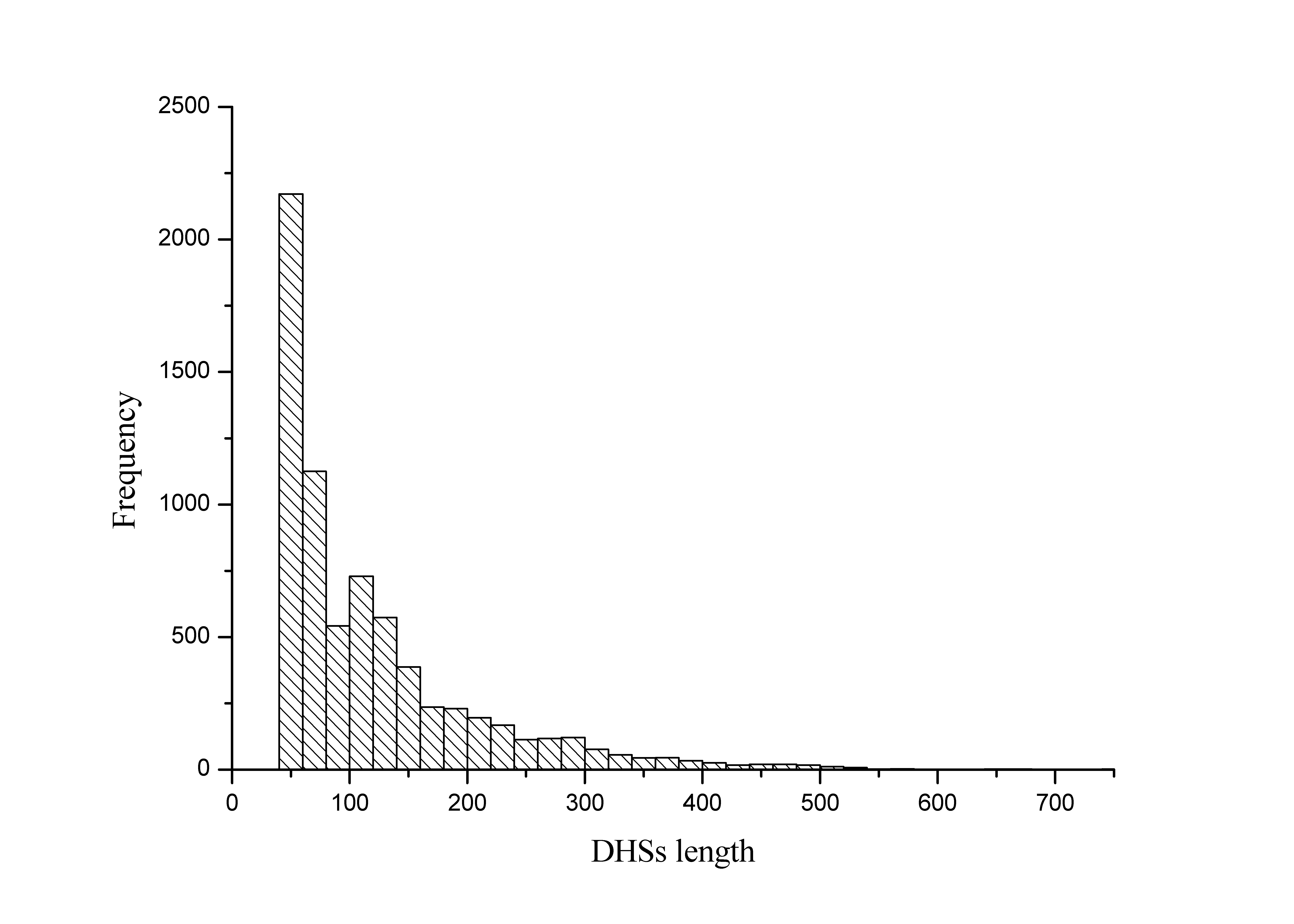


Supplementary Figure.S2 Distribution of DHS lengths

Supplemental Document 2:

*
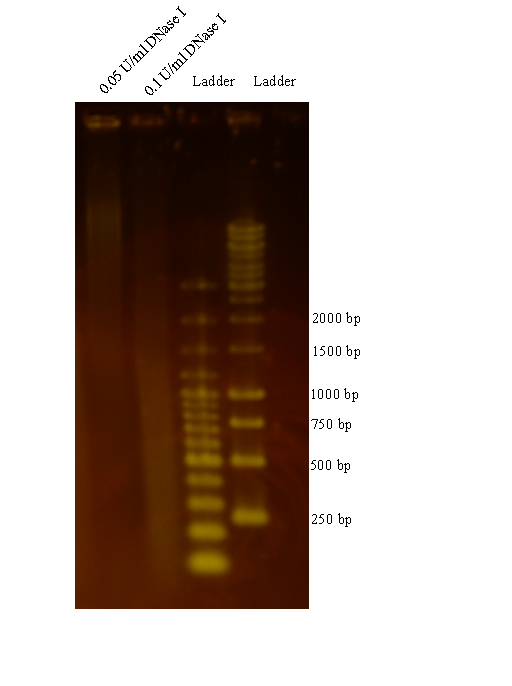
*

Supplementary Figure.S3 Gel electrophoresis of DNase I–digested naked DNA

Supplemental Document 3:


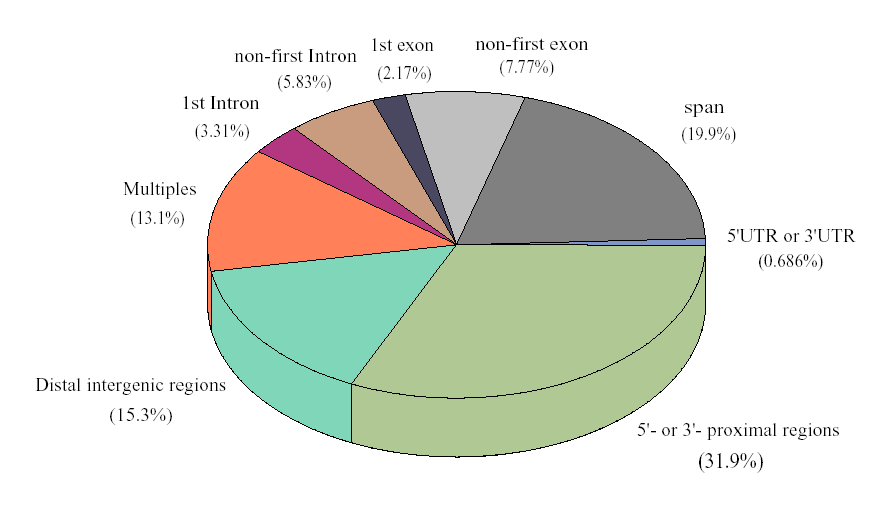


Supplementary Figure.S4 Genomic distribution of DHSs present in both samples.

“Proximal” and “nearby” have the same meaning, and refer to locations within 2 kb from the transcriptional start site (TSS) or transcription termination site (TTS) of the nearest coding genes. “Distal” intergenic locations correspondingly refer to locations more than 2 kb from a TSS or TTS. “Multiples” refers to DHSs located within loci annotated with more than one coding transcript, and “span” means DHSs spanning junctions between exons and introns.

Supplemental Document 4:

A Monte Carlo simulation was performed to determine the genomic distribution bias of the DHSs. In the simulation, which was repeated 1000 times, genomic regions corresponding to the DHSs in length, number and chromosomal distribution were randomly selected from the WormBase WS140 release of the *C. elegans* genome.

Supplemental Table. S1 The statistical analysis about Genomic distribution of all DHSs

| DHS genomic locations | Percentages of All DHSs | Statistical analysis | P-value |
| --- | --- | --- | --- |
| 5'- or 3'- proximal regions | 31.8% | Significantly enriched | P-value <0.005 |
| Distal intergenic regions | 15.6% | Slightly enriched | P-value <0.067 |
| Intragenic regions | 52.6% | Significantly depleted | P-value <0.001 |
| 5' and 3'UTR | 1.13% | Slightly enriched | P-value <=0.057 |
| First coding exon | 2.17% | Slightly enriched | P-value <=0.053 |
| Internal coding exon | 12.53% | Significantly enriched | P-value <0.001 |
| First Intron | 3.12% | Significantly depleted | P-value <0.001 |
| Internal Intron | 7.12% | Significantly depleted | P-value <0.001 |

It shows that *C. elegans* DHSs are enriched in intergenic regions, exons and UTRs, and are depleted in intragenic regions and introns.


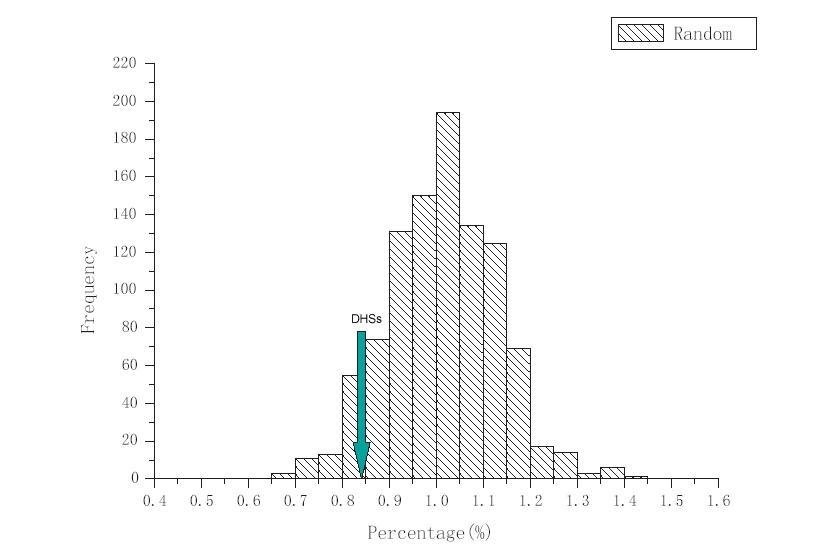


Supplementary Figure.S5 DHS frequency in the vicinity of known ncRNAs vs. the random sets (p-value <0.06)


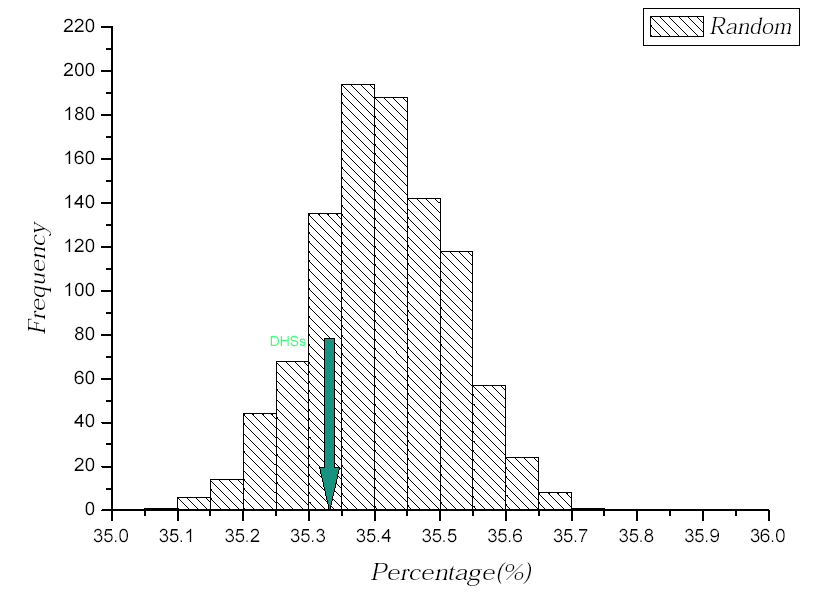


Supplementary Figure.S6 DHSs GC content vs. the random sets (p-value <0.205)


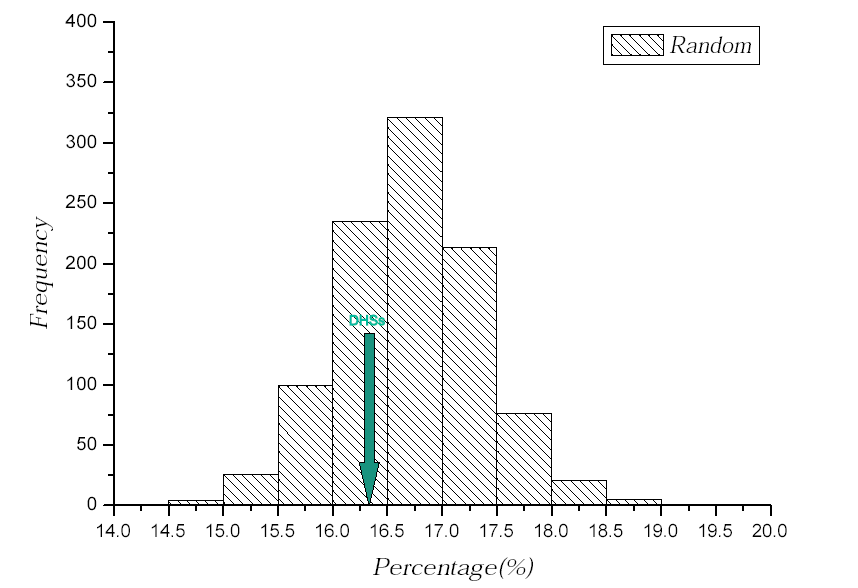


Supplementary Figure.S7 Fraction of DHS frequencies in operons relative to in intragenic regions vs. the random sets (p-value <0.267)

It indicates that there is no significantly statistical difference between operonic genes and non-operonic genes with respect to DHSs (i.e. no significant difference of the fraction of DHS frequencies in operons relative to in intragenic regions compared to the random sets).

Supplemental Table. S2 The statistical analysis about the conservation analysis of DHSs

| DHS genomic locations | Percentages of DHSs located in conserved regions | Statistical analysis | P-value |
| --- | --- | --- | --- |
| All DHSs | 47.62% | Significantly enriched | P-value <=0.001 |
| 5'- or 3'- proximal regions | 2.8% | No Statistical difference | P-value <=0.519 |
| Distal intergenic regions | 2.71% | Significantly enriched | P-value <0.038 |
| 5' or 3'UTR | 56.25% | No Statistical difference | P-value <0.135 |
| First coding exon | 56.49% | No Statistical difference | P-value <0.192 |
| Internal coding exon | 73.09% | Significantly enriched | P-value <0.001 |
| First Intron | 39.37% | Significantly depleted | P-value <0.021 |
| Internal Intron | 38.61% | Significantly depleted | P-value <0.001 |

Supplemental Document 5:

We found DHSs located within or proximal to 66 known ncRNAs including tRNAs, snoRNAs, microRNAs, snRNAs and snlRNAs.


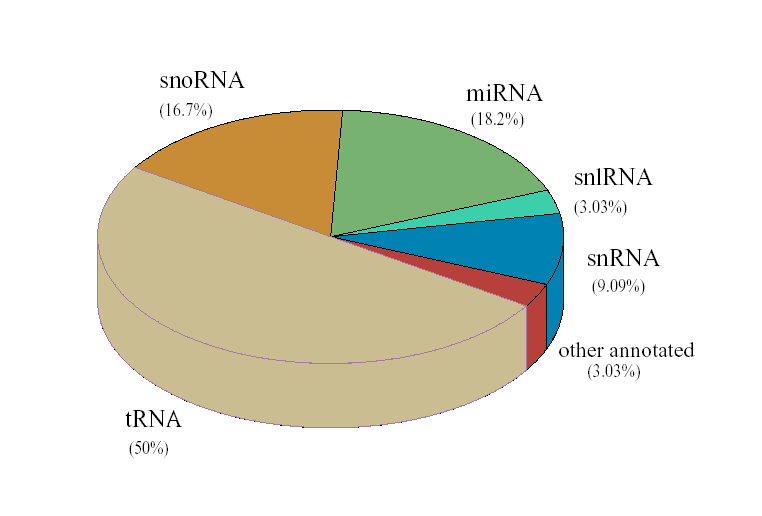


Supplementary Figure.S8 Fraction of known ncRNA classes located proximal to DHSs.


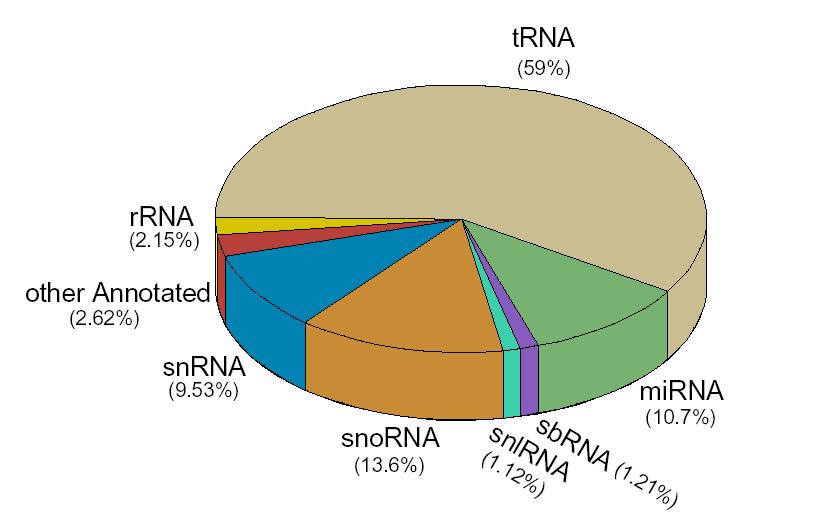


Supplementary Figure.S9 Fraction of known ncRNA classes in *C.elegans* genome.

Supplemental Document 6:

For the expression analysis, *C. elegans* gene expression datasets were obtained from the Genome B.C. *C. elegans* Gene Expression Consortium (http://elegans.bcgsc.bc.ca). These experiments were performed with the Affymetrix *C. elegans* GeneChip expression arrays. All microarrays were scaled to have an average signal intensity of 500. With a default p-value cut-off value 0.04, a coding gene was defined as expressed at the YA stage when showing a positive signal in all replicates at the young adult stage (based on WormBase release WS140).

Supplemental Table. S3 Coding genes expressed at the young adult stage

| Chromosomes | Number of Coding transcripts expressed at the YA stage | Fraction |
| --- | --- | --- |
| Chr I | 2212 | 56.6 % |
| Chr II | 2022 | 45.2% |
| Chr III | 2226 | 58.0% |
| Chr IV | 1958 | 46.5% |
| Chr V | 1946 | 33.4% |
| Chr X | 1516 | 41.8% |


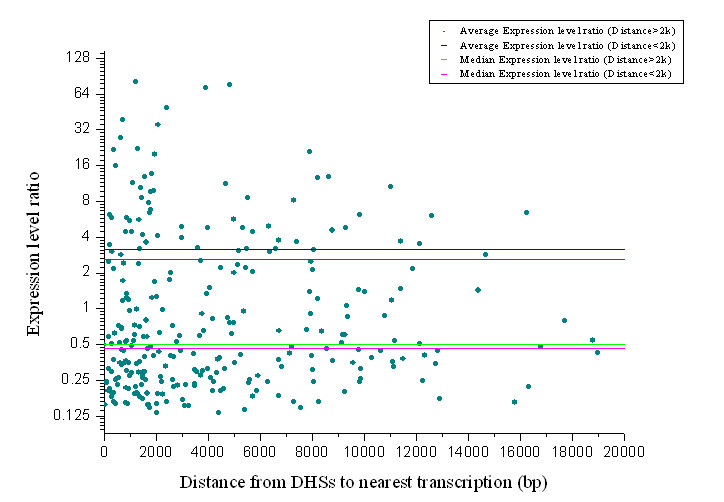


Supplementary Figure.S10 Relationship between DHS distance and nearest gene expression.

The figure shows the relationship between the distance between a DHS and its nearest gene, and the relative expression level of that gene. ("Expression level ratio" denotes the gene expression level divided by the average expression level of all coding genes at the young adult stage). Red and blue horizontal dashed lines mark the average expression level ratio of genes that have a nearby (<2 kb, blue) DHS versus genes with a more distant (>2 kb, red) DHS. Green and pink horizontal dashed lines mark the median expression level ratio of genes that have a nearby (<2 kb, blue) DHS versus genes with a more distant (>2 kb, red) DHS.


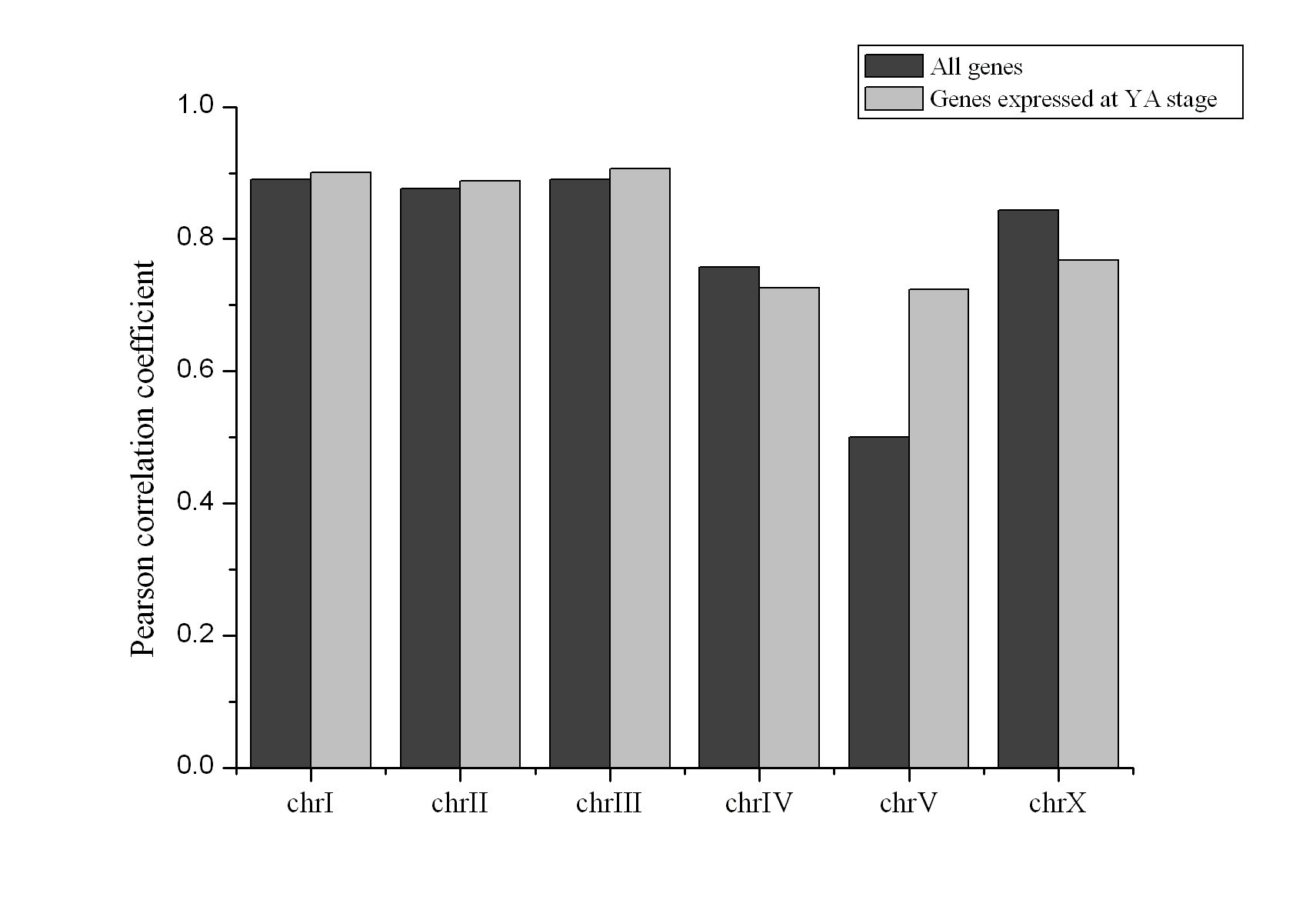


Supplementary Figure.S11 DHS distribution relative to gene specificity and expression

Relationship between distributions of DHSs and genes expressed at young adult stage. The Pearson correlation coefficients were calculated between the frequency of DHSs and YA genes in 0.5 Mb non-overlapping windows along each chromosome.

Supplemental Document 7:

The biotinylated Adaptor-I:

5’-Biotin-CCTCTCTATGGGCAGTCGGTGATTCCAAC-3’ 5’-GTTGGAATCACCGACTGCCCATAGAGA GG-3’

The second linker Adaptor-II:

5’-CGCTGCAGAGAATGAGGAACCCGGGGCAG-3’

5’-Amino-CTGCCCCGGGTTCCTCATTCTCTGCAGCG-3’

Tiling Array PCR primers:

Forward: 5’-TCTCTATGGGCAGTCGGTGAT-3’

Reverse: 5’-CTGCCCCGGGTTCCTCATTCTCT-3’
